# Genomic profiling and sites of metastasis in non-small cell lung cancer

**DOI:** 10.3389/fonc.2023.1212788

**Published:** 2023-09-12

**Authors:** Kok Hoe Chan, Arthi Sridhar, Ji Zheng Lin, Syed Hassan Raza Jafri

**Affiliations:** ^1^ Division of Hematology/Oncology, Department of Internal Medicine, McGovern Medical School at The University of Texas Health Science Center at Houston, Houston, TX, United States; ^2^ Department of Internal Medicine, McGovern Medical School at The University of Texas Health Science Center at Houston, Houston, TX, United States

**Keywords:** non-small cell lung cancer, NSCLC, metastasis, molecular profile, mutations, survival

## Abstract

**Background:**

We investigated the biological predisposition to site of metastasis in patients with NSCLC based on their molecular profiling and program death ligand PD-L1 status. We sought to identify any association between metastatic site and molecular profile in NSCLC patients.

**Methods:**

This was a retrospective analysis of patients with stage IV NSCLC who were newly diagnosed from January 2014 to June 2022. Clinical characteristics, pathology, molecular reports, and imaging were retrieved and analyzed.

**Results:**

A total of 143 patients were included in the study. Median age was 65 years, with an equal number of men (n=71) and women (n=72). The most common histology was adenocarcinoma (81.8%). At least one genetic mutation was discovered in 100 patients. Mutations with a targetable drug were found in 86 patients. The most common mutations were *TP53* (25.2%), *EGFR* (24.5%), *KRAS/NRAS* (20.3%), and *CDKN2A/2B* (7.7%). Patients with any mutation were significantly more likely to have metastatic disease to the brain (57% *vs*. 37%, p=0.03), but there was no difference in metastatic disease to bone (34% *vs*. 26%, p=0.32). Patients without a discoverable mutation were significantly more likely to have metastatic disease to other sites (e.g., adrenal gland 91% *vs*. liver 66%, p=0.002). There was no difference in progression-free survival (PFS) or overall survival (OS) between those with versus without mutations. Median PFS and OS were significantly longer in patients with an *EGFR* mutation than those with *KRAS/NRAS* or *TP53* mutations. Patients with PD-L1 >1% or *TP53* were significantly more likely to have metastatic disease to organs other than bone or brain (p=0.047 and p=0.023, respectively). We identified four prognostic groups in metastatic NSCLC. Patients with PD-L1 <1% and no actionable mutations have the poorest prognosis, with median survival of around 20 months.

**Conclusion:**

Patients with mutations discoverable on NGS are more likely to have metastatic disease to the brain. *KRAS/NRAS* in particular has a predilection to metastasize to the brain and bone. PD-L1 expression and a *TP53* mutation, on the other hand, tend to lead to metastasis of NSCLC to organs other than brain or bone. These results need to be corroborated in larger prospective studies.

## 1 Introduction

Non-small cell lung cancer (NSCLC) comprises about 85% of lung cancer. NSCLC is a term that includes a variety of different lung cancers, most notably adenocarcinoma, squamous cell carcinoma, and large-cell carcinoma. NSCLC also includes other subsets of lung cancer such as adeno-squamous carcinoma, sarcomatoid carcinoma, and non–small cell neuroendocrine tumors. Adenocarcinoma is the most common type of NSCLC (1). Since the introduction of next-generation sequencing (NGS), molecular genotyping has become essential in metastatic NSCLC, and the development of mutation-directed therapy has revolutionized the treatment of NSCLC. The overall prognosis for lung cancer continues to improve due to the evolving availability of targeted therapy and immunotherapy.

NSCLC can metastasize to adjacent tissues or organs by direct invasion or follow typical oncogenic metastasis by hematogenous, lymphatic or lympho-hematogenous pathway. NSCLC tends to metastasize to liver, brain, bone and adrenal glands. The process of cancer migration and metastasis is a sequential process, where cancer cells will either directly invade into surrounding tissues/organs or invade into vascular and lymphatic system and disseminate to other organs (2). Due to the complexity of lymphatic and vascular system, the metastatic pathway is always unpredictable. Hellman and Weichselbaum have once proposed that the probability and the sites of metastasis may reflect the inner state of tumor development in the process of acquiring necessity properties for dissemination (3). The discovery of molecular alterations especially in the metastasis/aggressive setting, may further revise the concept of oncogenic metastasis, highlighting the potential role of molecular alterations in directing the site of metastasis or dissemination. Cancer with mutations involving the epidermal growth factor receptor may have a distinct metastasis pattern highlighting the potential role of tumor genetics in the metastasis behavior of the tumor.

There is limited data to date to predict or assess the biological predisposition of site of metastasis in patients with NSCLC based on the molecular profiling. Kuijpers et al. reported in a retrospective analysis the potential association of molecular profiling of non-squamous NSCLC with the site of metastasis. They reported that about 54% of stage IV *EGFR*-positive tumors had bone metastasis at the time of diagnosis (4). Hsu et al. further highlighted a higher incidence of lung and brain metastasis in NSCLC patients with an *EGFR* mutation (5). Moreover, Hsu et al. also reported that the incidence of liver metastasis is significantly different between subtypes with the *EGFR* exon 19 deletion versus the exon 21 mutation, highlighting the potential association of molecular profiling in the spread of NSCLC tumors (5).

Based on these previous results, we propose that the biological predisposition to a metastatic site may differ between the molecular subgroups of NSCLC. Furthermore, differences in metastatic disposition could have a differential effect on morbidity, mortality, and the natural history of the disease. Hence, there is a paramount need to understand the association between the molecular landscape and metastatic site.

## 2 Materials and methods

### 2.1 Study subjects

This was a retrospective analysis conducted at UTHealth Houston/Memorial Hermann Cancer Center. All patients ≥18 years old with a newly diagnosis of stage IV primary NSCLC diagnosed between January 2014 and June 2022 were identified and retrieved. Additional inclusion criteria were pathology-proved NSCLC as per AJCC staging, 8th edition, available mutation analysis information, and sufficient medical information for analysis. Exclusion criteria included recent history of non-NSCLC cancer (i.e., any malignancy within 5 years before NSCLC diagnosis, except for skin tumors other than melanoma), non-invasive tumors, progressive NSCLC and tumors with molecular alterations identified on pathology material obtained ≥3 months after diagnosis. The database that we retrieved included patients’ clinical characteristics, medical history including medications, pathology report, molecular profile, imaging, and clinical laboratory data.

### 2.2 Ethical issues and informed consent

The study protocol was approved by the Institutional Review Board of both Memorial Hermann Hospital and The University of Texas Health Science Center at Houston. All procedures were conducted in compliance with the ethical standards of the Memorial Hermann Hospital, The University of Texas Health Science Center at Houston, and the Declaration of Helsinki for research on human beings. A waiver of HIPAA privacy authorization was been obtained through the Ethical Review Board.

### 2.3 Molecular profile and imaging methods

Biopsy results were obtained from either the primary lung tissue or metastatic site. Biopsies were reviewed and reports were authorized by a UTHealth pathologist. The molecular profile (NGS) results were obtained using commercially available assays (Foundation One or validated assays by UT Molecular Pathology). The NGS results were obtained from the initial biopsy tissue or metastatic site depending on original tissue sample availability. The choice of biopsy specimen (primary *vs* metastatic) chosen for NGS testing depended on 1) accessibility of tissue specimen for biopsy as well as 2) amount of tissue available to run NGS. For patients who did not provide a tissue sample for NGS testing, molecular profiles were obtained from peripheral blood. As for the PD-L1, the PD-L1 expression was determined by the Tumor Proportion Score and classified into TPS <1%, TPS 1 to 49% and TPS ≥50%.

### 2.4 Metastatic lesions evaluation

The evaluation of the metastatic lesions was conducted based on imaging either via PET/CT scan, CT chest abdomen pelvis with contrast, MRI brain with/without contrast and or biopsy of the site of metastasis. There are about 59% of the patients in our study have the biopsy obtained from the distant metastatic sites. The assessment of presence of metastatic disease was first done by the treating physician and later by our research team giving a second layer of confidence in existence of metastatic disease

### 2.5 Statistical analysis

We analyzed the patients’ demographic characteristics, as well as clinical and biochemical data. Descriptive data were represented by percentages, mean ± standard deviation, medians, and numbers. Continuous-variable analysis was performed with Kruskal–Wallis one-way analysis of variance and t-test for non-normal and normal distribution, respectively. As for categorical variables, the χ2 or Fisher’s exact test was used. Progression-free survival (PFS) was calculated from the time of initial diagnosis until the time of disease progression. Overall survival (OS) was calculated from the time of initial diagnosis until censoring (death/events or date of last follow-up). PFS and OS were analyzed using the Kaplan–Meier test.

We did all data analysis using the statistical software GraphPad Prism version 14.0.2. Statistical significance was achieved if the null hypothesis could be rejected at P < 0.05 with a 95% confidence interval (CI). We also compared differences in clinical parameters, mutation profile, PFS, OS, and other parameters in patients who with OS <12 months versus OS ≥12 months.

## 3 Results

### 3.1 Clinical characteristics and demographics

**Figure 1:**
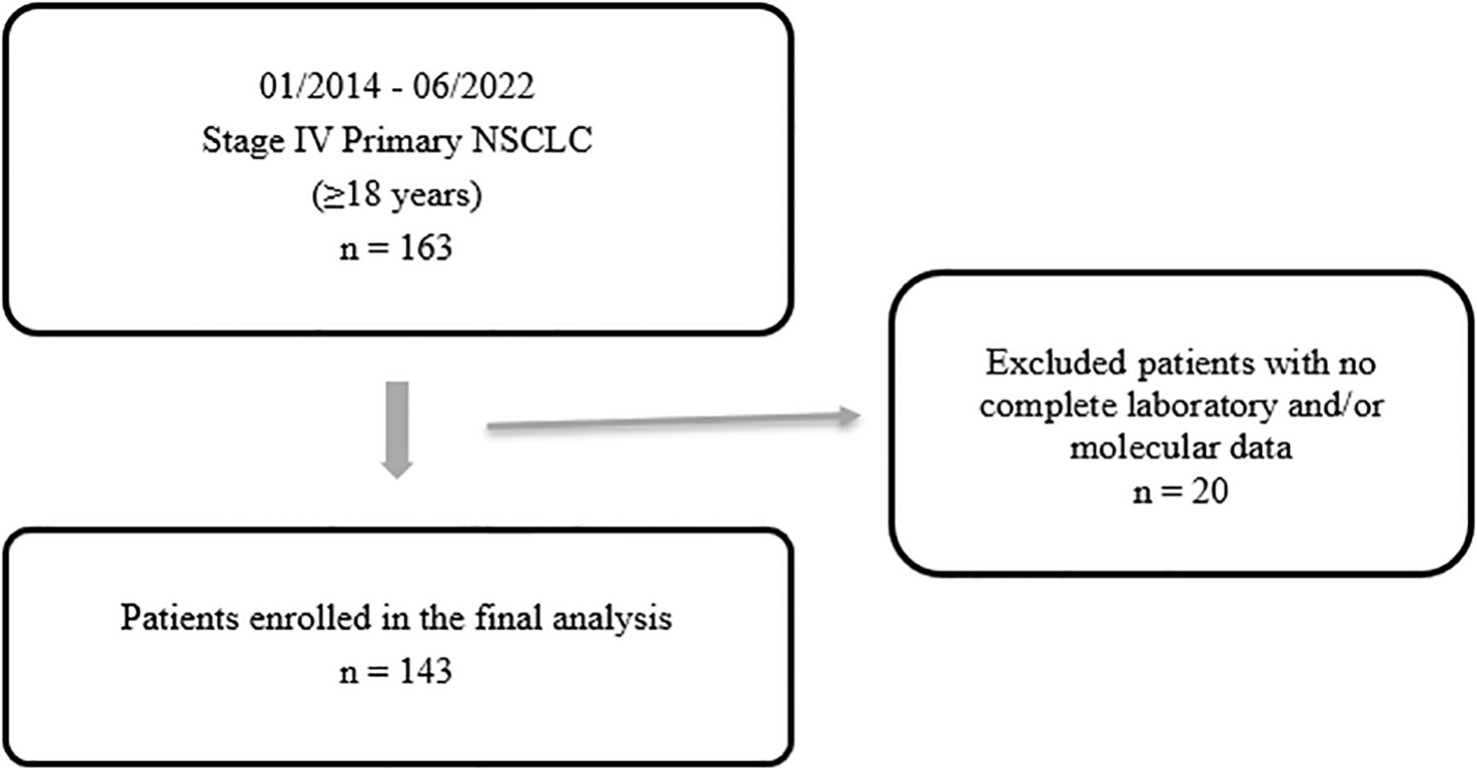
Diagram of study participants eligibility.

After excluding patients who did not have complete clinical data, a total of 143 patients were included, and their NGS panels were analyzed (**Figure 1**). Fifty-seven (40%) NGS reports were obtained from primary lung tissue, 84 (59%) from metastatic sites, and 2 (1%) from peripheral blood (liquid biopsy). Median age was 65 years, with an equal number of men (n=71) and women (n=72). The most common histology was adenocarcinoma (81.8%), followed by squamous cell cancer (11.9%) and large cell carcinoma (3.5%). At least one genetic mutation was discovered in 100 patients (70%); 43 (30%) had no discoverable genetic mutation. Mutations with a targetable drug were found in 86 patients (60%), and many patients had >1 genetic mutation. The most common mutations were *TP53* (25.2%), *EGFR* (24.5%, 63% with the classic targetable *EGFR* mutation exon 19 and L858R and 37% with atypical *EGG*R mutations such as exon 18, exon 20, L861Q, or T790M), *KRAS/NRAS* (20.3%), and *CDKN2A/2B* (7.7%). The most common metastatic sites were brain (51%), bone (31.5%), contralateral lung (23.1%), pleura (21%), and adrenal gland (14%). Program death ligand PD-L1 status was known in 117 (81.8%) patients. Of these, 45 (38%) had no PD-L1 expression, 41(35%) had 1%–49%, and 31 (21.7%) had >50% PD-L1 expression.

The median PFS and OS for entire cohort were 24 months and 41.2 months, respectively. Detailed clinical characteristics and demographic analysis are presented in **Table 1**.

**Table 1:** Clinical characteristics and demographics.

	N=143
Age, (median range]) years	65 (32–92)
Sex	
Male	71 (49.7)
Female	72 (50.3)
Race	
White	51 (35.7)
Black	49 (34.3)
Hispanic	5 (3.5)
Asian	23 (16.1)
Other	15 (10.5)
Types of NSCLC	
Adenocarcinoma	117 (81.8)
Squamous cell carcinoma	17 (11.9)
Large-cell carcinoma	5 (3.5)
Others, n (%)	4 (2.8)
Targeted mutation profile	
*EGFR*	35 (24.5)
*KRAS/NRAS*	29 (20.3)
*MET*	6 (4.2)
*HER2*	5 (3.5)
*ALK*	4 (2.8)
*BRAF*	4 (2.8)
*RET*	2 (1.4)
*NTRK*	1 (0.7)
*ROS*	0
Other mutations	
*TP53*	36 (25.2)
*CDKN2A/2B*	11 (7.7)
*STK11*	9 (6.3)
*PIK3CA*	5 (3.5)
*FGFR*	4 (2.8)
*BRCA1/2*	2 (1.4)
PD-L1 status	
<1%	45 (31.5)
1%–49%	41 (28.7)
–50%	31 (21.7)
Site of metastasis	
Brain	73 (51.0)
Bone	45 (31.5)
Contralateral lung	33 (23.1)
Pleura	30 (21.0)
Adrenal gland	20 (14.0)
Liver	14 (9.8)
Other	10 (7.0)
PFS, median (range) months	24.0 (0.2–121.1)
OS, median (range) months	41.2 (0.9–138.6)

Data are no. (%) unless otherwise given.

### 3.2 Mutations and sites of progression or metastatic disease

Patients with any mutation were significantly more likely to have metastatic disease to the brain (57% *vs*. 37%, p=0.03), but there was no difference in rates of metastatic disease to the bone (34% *vs*. 26%, p=0.32). Patients without a discoverable mutation were significantly more likely to have metastatic disease to other sites (e.g., adrenal gland or liver; 91% *vs*. 66% for patients with discoverable mutations, p=0.002). In our study NGS testing was done on primary site (40%), metastatic (59%), and peripheral blood (1%). Branched evolution of tumor during the progression of disease has been reported to result in intratumor heterogeneity, nonetheless, single site NGS can accurately estimates genetic landscape of the tumor. To verify the validity of our results, we have performed a stratified analysis of mutations and sites of metastatic disease in biopsy obtained from primary and metastatic lesions. Based on this stratified analysis, there was no difference in sites of metastatic disease predicted based on presence of absence of discoverable mutation (**Supplementary Table**).

When stratifying based on the mutational profile, patients with TP53 mutations were significantly more likely to have metastatic disease to organs other than bone or brain (hazard ratio [HR] 0.29, p=0.023). With regard to single-organ metastasis, tumors with *KRAS/NRAS* mutations had a predilection to metastasize to brain and bone, (45% of patients, HR 3.47, p=0.04). A detailed analysis is given in **Table 2**. Tumors with *EGFR* and *KRAS/NRAS* mutations had a predilection for brain metastasis, which was observed in 60% and 59% of patients, respectively.

**Table 2:** Site of metastasis or progression in patients with and without mutations.

Site of metastasis	Any mutation (n=100)	No mutation (n=43)	OR	95% CI (p value)
Brain, n (%)	57 (57)	16 (37)	2.24	1.06–4.73 (p=0.03)
Bone, n (%)	34 (34)	11 (26)	1.50	0.69–3.20 (p=0.32)
Other organs, n (%)	66 (66)	39 (91)	0.20	0.07–0.59 (p=0.002)
PFS, median (range) months	24.0 (0.2–92.3)	22.9 (0.9–121.1)	Log-rank p=0.58	
OS, median (range) months	41.2 (1.1–138.6)	36.7 (0.9–121.1)	Log-rank p=0.71	

**Figure 2:**
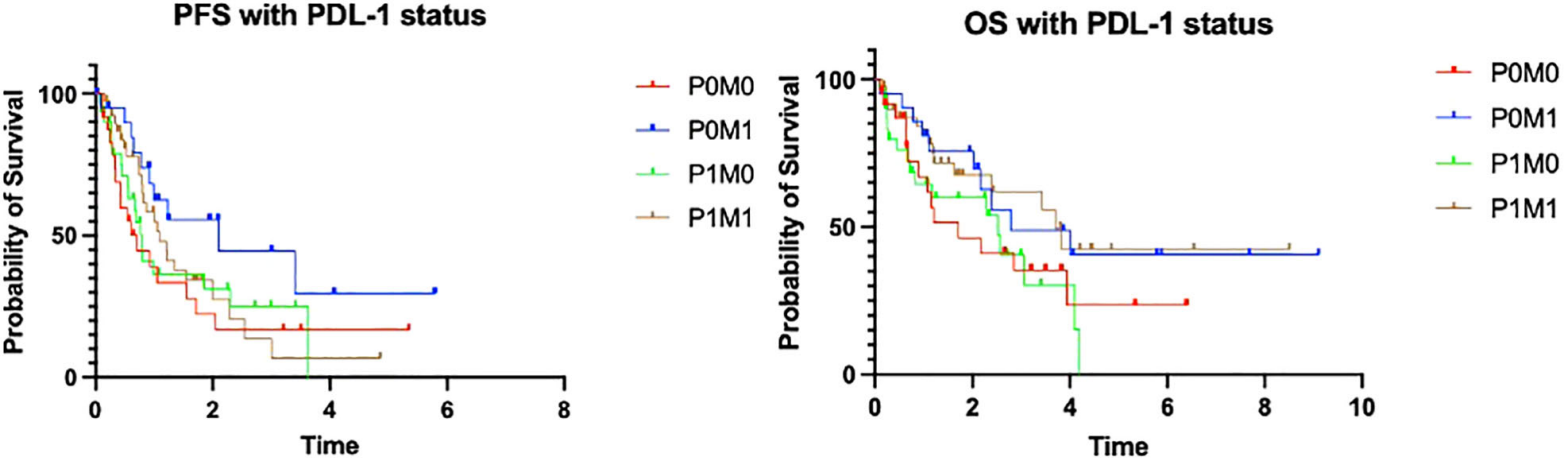
PFS and OS of patients with and without mutations.

When comparing the survival outcomes (PFS and OS) in patients with or without mutations, there was no difference observed in PFS (24 *vs*. 22.9 months, respectively, log rank p=0.58) or OS (41.2 *vs*. 36.7 months, respectively, log rank p=0.71) between these two groups via Kaplan–Meier analysis (**Figure 2**). It might be expected that the survival outcome is better in the subgroup of patients with mutations in light of targeted therapies; however, approximately 45% of patients had either *TP53* or *KRAS* mutations, which have been shown in multiple studies to indicate poorer outcomes in NSCLC.

### 3.3 Survival outcome and targeted mutations

**Figure 3:**
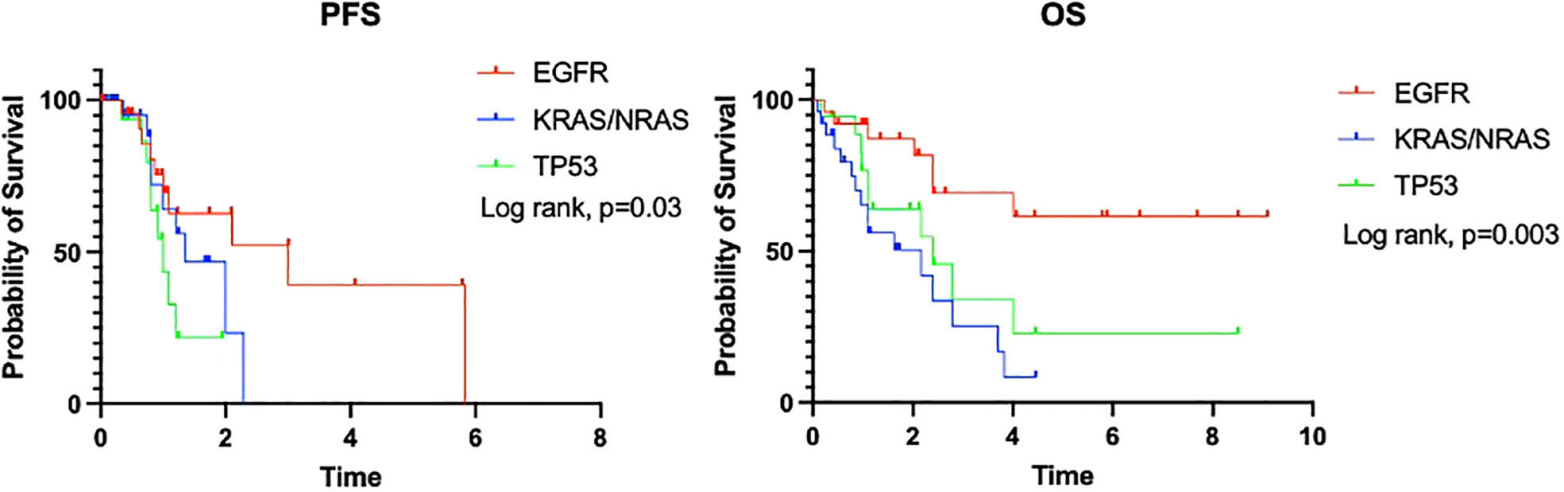
PFS and OS of patients by targeted mutational profile.

Median PFS was significantly longer in patients with an *EGFR* mutation than those with *KRAS/NRAS* or *TP53* mutations (36 *vs*. 16.2 *vs*. 11.9 months, p=0.03). Median OS for patients with an *EGFR* mutation was not reached and was significantly longer than in patients with *TP53* (28.7 months) or *KRAS/NRAS* (26 months) mutations (p=0.003) (**Figure 3**).

### 3.4 PD-L1 and sites of progression or metastatic disease

With regard to PD-L1 status, patients with PD-L1 >1% were significantly more likely to have metastatic disease to organs other than bone or brain (HR 0.4, p=0.047). A detailed analysis is shown in **Table 3**.

**Table 3:** Comparison of site of metastasis/progression between patients with PD-L1 <1% *vs*. PD-L1 ≥1%.

Site of metastasis	PD-L1 <1% (n=45)	PD-L1 ≥1% (n=72)	OR	95% CI (p-value)
Brain, n (%)	19 (42)	37 (51)	0.69	0.32–1.43 (p=0.33)
Bone, n (%)	13 (29)	21 (29)	0.97	0.42–2.32 (p=0.97)
Other organs, n (%)	32 (71)	62 (86)	0.40	0.14–0.97 (p=0.047)

### 3.5 Prognostic stratification and outcome of metastatic NSCLC based on activating mutation profile and PD-L1 status

**Figure 4:**
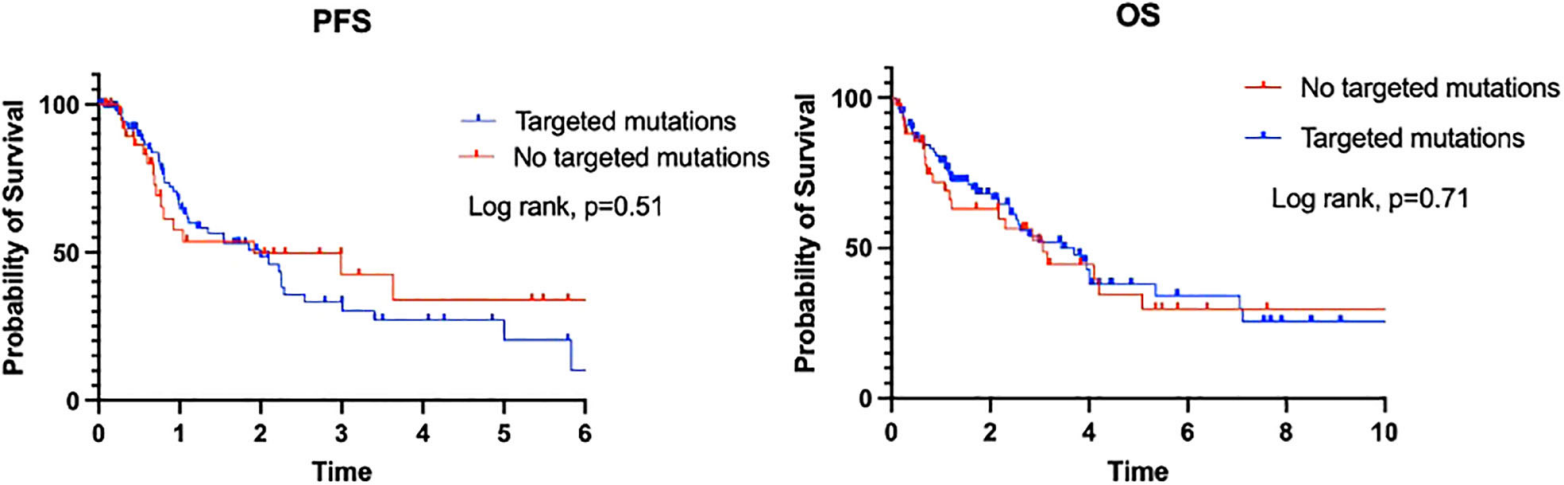
PFS and OS of patients with regard to PD-1 status.

When we compared the PFS and OS with regard to PD-L1 status and the presence of a discoverable mutation, 4 prognostic groups were identified: P0M0 (PD-L1 <1% and no actionable mutation), P0M1 (PD-L1 <1% with an actionable mutation), P1M0 (PD-L1 ≥1% and no actionable mutation), and P1M1 (PD-L1 ≥1% with an actionable mutation) (**Table 4**). The P0M0 subgroup (n=24) had the worst median PFS and OS (8.5 and 20.6 months, respectively). The P1M1 subgroup (n=41) had a median PFS of 13.1 months and the longest OS of 44.5 months. Patients in the P0M1 group had a longer PFS than those in the P1M1 group (25.2 *vs*. 13.1 months), likely due to the presence of more patients with an *EGFR* mutation (57% *vs*. 32%) However, this did not translate into improved OS (P0M1: 33.5 months, P1M1: 44.5 months, log-rank p=0.98), perhaps because of the durable long-term response to immunotherapy in patients with PD-L1 ≥1% or due to limited second-line options in P0M1 patients at the time of progression. The results were not significant, likely because of the small sample size in each category. Detailed survival analysis depicted in **Figure 4** and **Table 4**.

**Table 4:** Comparison of site of survival outcome between patients PD-L1 <1% *vs* PD-L1 >=1%, stratified by presence of targeted mutations.

Median (range) survival, months	P0M0 (n=24)	P1M0 (n=31)	P0M1(n=21)	P1M1 (n=41)
PFS	8.5 (1.3–64.2)	9.5 (0.9–43.6)	25.2 (0.2–92.3)	13.1 (0.6–58.3)
OS	20.6 (1.3–76.8)	30.3 (0.9–50.4)	33.5 (1.1–109.3)	44.5 (1.9–102.2)

### 3.6 Survival and clinical parameters

When comparing patients with an OS >12 months to those with OS ≥12 months, younger age, adenocarcinoma subtype, the presence of an *EGFR* mutation, and contralateral lung metastasis were associated with longer OS (p-value 0.014, <0.01, 0.02, and 0.019, respectively).

By contrast, Black patients, those with squamous cell carcinoma, the presence of an *HER2* mutation, and brain or bone metastasis were associated with poorer OS (p-value <0.01, 0.023, 0.028, 0.034, and 0.039, respectively). A detailed analysis is shown in **Table 5**.

**Table 5:** Comparison of clinical parameters between patient with OS <12 months *vs*. ≥12 months.

Characteristic	OS <12 months (n=49)	OS ≥12 months (n=94)	p-value
Age, median (range) years	68 (51–90)	63.5 (32–92)	***0.0140***
Sex			
Male	28 (57)	43 (46)	0.2203
Female	21 (43)	51 (54)	
Race			
White	18 (37)	33 (35)	0.8470
Black	24 (49)	25 (27)	***0.0074***
Hispanic	1 (2)	4 (4)	0.4939
Asian	4 (8)	19 (20)	0.0627
Other	2 (4)	13 (14)	0.0710
Type of NSCLC			
Adenocarcinoma	35 (71)	82 (87)	***0.0200***
Squamous cell carcinoma	10 (20)	7 (7)	***0.0230***
Large-cell carcinoma	2 (4)	3 (3)	0.7833
Other	2 (4)	2 (2)	0.5012
Targeted mutations			
*EGFR*	7 (14)	28 (30)	***0.0407***
*KRAS/NRAS*	14 (29)	15 (16)	0.0750
*MET*	1 (2)	5 (5)	0.3534
*HER2*	4 (8)	1 (1)	***0.0283***
*ALK*	0	4 (4)	0.1430
*BRAF*	1 (2)	3 (3)	0.6921
*RET*	0	2 (2)	0.3038
*NTRK*	0	1 (1)	0.4687
*ROS*	0	0	
Other mutations			
*TP53*	13 (27)	23 (24)	0.7874
*CDKN2A/2B*	6 (12)	6 (6)	0.2302
*STK11*	5 (10)	3 (3)	0.0833
*PIK3CA*	2 (4)	3 (3)	0.7833
*FGFR*	1 (2)	3 (3)	0.6921
*BRCA1/2*	1 (2)	1 (1)	0.6368
PD-L1 status			
<1%	15 (31)	30 (32)	0.8735
1%–49%	19 (39)	22 (23)	0.0537
≥50%	9 (18)	22 (23)	0.4878
Site of metastasis			
Brain	27 (55)	34 (36)	***0.0339***
Bone	21 (43)	24 (26)	***0.0388***
Contralateral lung	5 (10)	26 (28)	***0.0185***
Pleura	8 (16)	22 (23)	0.3905
Adrenal gland	10 (20)	10 (11)	0.1305
Liver	6 (12)	8 (9)	0.5563
Other	5 (10)	5 (5)	0.3111

Data are no. (%) unless otherwise indicated. P values in bold italic reached statistical significance.

## 4 Discussion

This study has demonstrated varying results with respect to the common sites of metastasis for NSCLC patients with common mutations. *EGFR* mutation can be seen in up to 10% of white patients with NSCLC and up to 50% in NSCLC patients of Asian origin (6). In our study, *EGFR* was the most common actionable mutation seen, accounting for about 24% of all mutations. *KRAS* gene mutations accounted for about 25% of driver mutations, and other targetable activating gene mutations found included *EML4-ALK, HER-2, RET, MET, and ROS1*, among others (7). Classic *EGFR* mutations (exon 19 deletion and L858R) have been shown to be commonly associated with bone and pleural metastasis (4). Another single-institution study showed that *EGFR* exon 19 deletions were commonly associated with bone and intrapulmonary metastasis. That study also showed that patients with a T790M mutation had higher incidences of brain, bone, and liver metastasis compared with patients who had other *EGFR* gene mutations (8). In contrast, another retrospective study analyzing data from 105 patients did not find any statistically significant difference in the site of metastasis for *EGFR* mutant *vs* wild-type tumors (9). We also found that patients with a mutation were significantly more likely to have metastatic disease to the brain. Tumors with *EGFR* and *KRAS/NRAS* mutations specifically had a predilection for brain metastasis, which was observed in 60% and 59% of patients, respectively.

Our data showed that patients with an *EGFR* gene mutation had a longer OS than patients without this mutation or patients with a *KRAS* or *TP53* mutation. This finding is consistent with existing literature describing a longer OS for patients with an activating *EGFR* mutation or those who received an *EGFR*-TKI (10). The original IPASS study was able to show improved 12-month PFS in patients who received an *EGFR*-TKI compared to patients who received chemotherapy (24.9% *vs*. 6.7%) (11). In the FLAURA trial, a newer generation *EGFR*-TKI, osimertinib, showed improved PFS compared to previous generations (HR for progression or death 0.46) and, for the first time, PFS also translated to an increase in OS (HR 0.8, 95% CI 0.64–1.00) (12). We also found a significantly decreased 12-month OS in patients with a *HER-2* mutation, which is consistent with previous studies associating *HER-2* mutation with a poor prognosis (13). Furthermore, although some studies have found shorter PFS and OS in *KRAS*-mutated tumor compared to non–*KRAS*-mutated tumors, we did not find a difference in 12-month OS by *KRAS* mutation status (14, 15).

*TP53* is known to be the most frequent mutation found in NSCLC, and this was also demonstrated in our study (16). An analysis of 1441 patients with NSCLC showed that *TP53* mutations are not only the most common mutation found but also that a *TP53* gene mutation is an adverse prognostic factor (16). In our study, patients with a *TP53* mutation had a significantly poorer OS and PFS than patients with *EGFR* mutations; however, this result was not statically significant, likely due to our small sample size. Recent data also suggest that activated *TP53* can be associated with *EGFR* mutations and can be utilized in the stratification of tumor types (16). Studies have shown that these co-mutations are associated with a poorer prognosis (16, 17). Recent data also suggest that *TP53* mutations sensitize chemo-resistant tumors with *EGFR*-activating mutations (16). In our retrospective analysis, only 8 patients had both *EGFR* and *TP53* mutations, although their PFS was shorter than that of patients with an *EGFR* mutation alone (10.4 *vs*. 15 months, respectively), and there was no difference in OS (29.4 *vs*. 29.1 months, respectively). The data was not statistically significant due to our small sample size.

PD-L1 plays an important role in the immune checkpoint responsible for allowing tumor cells to evade the immune system. Currently, several monoclonal therapies are available that target tumors displaying the appropriate biomarkers. Thus, PD-L1 remains a clinically significant biomarker for the treatment of NSCLC. However, associations between the presence of PD-L1 markers and clinicopathologic features of NSCLC have not been well studied. We found no significant rate of metastatic disease to the brain or bone in patients with PD-L1 >1% compared to those with PD-L1 <1%, but we did find a significant difference in rates of metastatic disease to other organs. This is aligned with the results of a study by Zhang et al., which found an association between PD-L1 expression and abdominal organ metastasis but not brain metastasis (18). However, another study by Lin et al. found no significant correlation between PD-L1 expression and metastasis to the brain or other organs (19). Furthermore, there are mixed results on whether PD-L1 expression has any association with lymph node metastasis: some studies have shown that it does, while others have found the opposite results (20, 21). Overall, the role of PD-L1 expression and its correlation to metastatic sites is unclear due to the limited number of studies available, and further research is needed to determine its use as a prognostic factor.

In a systemic literature review by Brody et al., out of 10 studies, 4 reported a significantly shorter survival with higher PD-L1 expression, and 2 reported shorter survival with higher PD-L1 expression that was either not significant or not analyzed (22). In the same review, 3 studies found no significant association between PD-L1 levels and survival, and 1 study reported longer survival with higher levels of PD-L1 expression (22). In our study, we found a nonsignificant increase in OS associated with PD-L1 between the P0M0 group and the P1M0 group (20.6 *vs*. 30.3 months) and between the P0M1 group and the P1M1 group (33.5 *vs*. 44.5 months). The longer OS in the P1M1 groups was likely due to more targeted therapy options.

Based on our data, we identified 4 prognostic groups in metastatic NSCLC based on the current treatment paradigm. Patients with no PD-L1 expression (<1%) and no discoverable mutation had the worst outcomes, whereas patients with the presence of a targetable mutation and PD-L1 >1% had the best outcomes. These results need to be validated in larger studies to see whether they hold true.

There are a few strengths of this study. First, we not only analyzed molecular driver genes but also included PD-L1 expression levels in identifying the potential site of metastasis and its role in survival outcomes in patients with stage IV NSCLC. We also gathered data on non-targetable molecular mutations, including *HER2, ALK, ROS, BRAF, NTRK, RET, CDKN2A*, and others. Furthermore, up to 60% of patients had PET/CT scan either at the time of diagnosis or at progression, which allowed better detection of metastasis compared with CT scan alone.

Our study has a few limitations. First, this is single-center retrospective study, which may limit the generalizability of the results; however, our findings are consistent with those of other large population studies. Second, we have a small patient sample for each molecular driver cohort, which makes the analysis and interpretation difficult; thus, no definitive comparison or conclusions can be drawn for each of the cohorts. For example, we had only 4 patients with *HER-2* mutations and only 8 patients with both *TP53* and *EGFR* mutations. Third, there may be a bias due to the site of metastasis, given that we included the molecular profile obtained from primary and metastasis samples. There were also two liquid biopsies included in the analysis. Lastly, our study only includes patients with *de novo* Stage IV NSCLC and trying to identify the pattern of mutation in respect to site of metastasis. In our study, we reported the incidence and predilection of site of metastasis with the molecular profile, we were not able to use the molecular profile to predict the site of progression which require different research methodology.

## 5 Conclusion

This retrospective analysis provides greater insight into the correlation between site of metastasis and prognosis of patients with stage IV NSCLC and their molecular profiles and PD-L1 status. Patients with discoverable mutations on NGS are more likely to have metastatic disease to the brain. Tumors with *KRAS/NRAS* mutations in particular showed a predilection to metastasis to the brain and bone and were associated with poorer prognosis. Patients with *EGFR* mutations, despite these tumors having a great propensity to brain metastasis, have significantly better PFS and OS than patients with *KRAS/NRAS* and *TP53* mutations, likely due to targeted therapy options. PD-L1 expression and TP53 mutation, on the other hand, tend to lead to disease metastatic to organs other than brain or bone. Our data suggested that four prognostic groups can be identified in metastatic NSCLC based on current treatment paradigm. Patients with PD-L1 <1% and no actionable mutations have the poorest prognosis, with a median survival of around 20 months. This is an important example of using real-world data to predict survival outcomes of patients with stage IV NSCLC, and these results need to be corroborated in larger studies.

## Data availability statement

The original contributions presented in the study are included in the article/**Supplementary Material**. Further inquiries can be directed to the corresponding author.

## Ethics statement

The study protocol was approved by the Institutional Review Board of both Memorial Hermann Hospital and The University of Texas Health Science Center at Houston. The studies were conducted in accordance with the local legislation and institutional requirements. The ethics committee/institutional review board waived the requirement of written informed consent for participation from the participants or the participants’ legal guardians/next of kin because This is a retrospective cohort analysis.

## Author contributions

KC contributed to the conception ad design, acquisition, analysis, and interpretation of data, as well as participated in drafting and revision of the manuscript. AS and JL actively participated in collecting data, analysis of data, and drafting and revision of manuscript. SJ contributed to idea design, data analysis, and critically revision of the manuscript and as well as approving the final version of manuscript. All authors contributed to the article and approved the submitted version.
