# Supplementary material for: Genomic profiling and sites of metastasis in non-small cell lung cancer
Source: Front Oncol. 2023 Sep 12;13:1212788. doi: 10.3389/fonc.2023.1212788 (PMC10523019; doi:10.3389/fonc.2023.1212788)
Supplement: Supplementary file 1 [file Table_1.docx]

Supplementary Table 1: Acronyms used in this manuscript.

| Abbreviation | Definition |
| --- | --- |
| NSCLC | Non-small cell lung cancer |
| PD-L1 | Program death ligand 1 |
| *TP53* | Tumor protein p53 |
| *EGFR* | Epidermal growth factor receptor |
| *KRAS* | Kirsten rat sarcoma virus |
| *NRAS* | Neuroblastoma RAS viral oncogene homolog |
| *CDKN2A/2B* | Cyclin-dependent kinase inhibitor 2A/2B |
| *HER-2* | Human epidermal growth factor receptor 2 |
| *MET* | Mesenchymal epithelial transition |
| *RET* | Rearranged during transfection |
| *BRAF* | v-raf murine sarcoma viral oncogene homolog B1 |
| *NTRK* | Neurotrophic tyrosine receptor kinase |
| TKI | Tyrosine kinase inhibitor |
| PFS | Progression free survival |
| OS | Overall survival |
| HR | Hazard ratio |
| CI | Confidence interval |
| NGS | Next generation sequencing |
| UTHealth | The University of Texas Health Science Center at Houston |
| PET/CT | Positron Emission Tomography and Computed Tomography |
| CT | Computed Tomography |
| SUV | Standardized uptake value |
| SUVmax | Maximum standardized uptake value |

Supplementary Table 2: Clinical characteristics and demographics

|  | N=143 | Biopsy from the Primary, n=57 | Biopsy from the Metastasis, n=84 | Liquid Biopsy, n=2 |
| --- | --- | --- | --- | --- |
| Targeted mutation profile |  |  |  |  |
| *EGFR* | 35 (24.5) | 12 | 22 | 1 |
| *KRAS/NRAS* | 29 (20.3) | 11 | 18 | 0 |
| *MET* | 6 (4.2) | 2 | 3 | 1 |
| *HER2* | 5 (3.5) | 3 | 2 | 0 |
| *ALK* | 4 (2.8) | 0 | 4 | 0 |
| *BRAF* | 4 (2.8) | 0 | 4 | 0 |
| *RET* | 2 (1.4) | 1 | 1 | 0 |
| *NTRK* | 1 (0.7) | 0 | 1 | 0 |
| *ROS* | 0 | 0 | 0 | 0 |
| Other mutations |  |  |  |  |
| *TP53* | 36 (25.2) | 14 | 22 | 0 |
| *CDKN2A/2B* | 11 (7.7) | 5 | 6 | 0 |
| *STK11* | 9 (6.3) | 3 | 6 | 0 |
| *PIK3CA* | 5 (3.5) | 3 | 2 | 0 |
| *FGFR* | 4 (2.8) | 2 | 2 | 0 |
| *BRCA1/2* | 2 (1.4) | 2 | 0 | 0 |
| Site of metastasis |  |  |  |  |
| Brain | 73 (51.0) | 32 | 40 | 1 |
| Bone | 45 (31.5) | 19 | 25 | 1 |
| Contralateral lung | 33 (23.1) | 12 | 21 | 0 |
| Pleura | 30 (21.0) | 12 | 17 | 1 |
| Adrenal gland | 20 (14.0) | 6 | 14 | 0 |
| Liver | 14 (9.8) | 8 | 5 | 1 |
| Other | 10 (7.0) | 5 | 5 | 1 |

Supplementary Table 3: Site of metastasis or progression in patients with and without mutations, stratified by the biopsy site, biopsy obtained from primary site of disease

| Site of metastasis | Any mutation (n=37) | No mutation (n=20) | OR | 95% CI (p value) |
| --- | --- | --- | --- | --- |
| Brain, n (%) | 23 (62) | 9 (45) | 2.00 | 0.67–5.82  (p=0.21) |
| Bone, n (%) | 15 (41) | 4 (20) | 2.72 | 0.72–8.54 (p=0.12) |
| Other organs, n (%) | 25 (68) | 18 (90) | 0.23 | 0.05–1.03 (p=0.06) |

Supplementary Table 4: Site of metastasis or progression in patients with and without mutations, stratified by the biopsy site, biopsy obtained from metastatic site of disease

| Site of metastasis | Any mutation (n=63) | No mutation (n=23) | OR | 95% CI (p value) |
| --- | --- | --- | --- | --- |
| Brain, n (%) | 34 (54) | 7 (30) | 2.68 | 0.99-6.80 (p=0.053) |
| Bone, n (%) | 19 (30) | 7 (30) | 0.98 | 0.35–2.57 (p=0.98) |
| Other organs, n (%) | 41 (65) | 21 (91) | 0.18 | 0.04–0.75 (p=0.02) |
